# Evaluation of automated microscopy sediment analysis in urinary tract infection screening: a practical insight in adjusting fixed cut-off values for urine culture

**DOI:** 10.1007/s11255-023-03654-6

**Published:** 2023-06-05

**Authors:** Elke Bovelander, Maarten Raijmakers, Dick van Dam, Yvette Kraat, Chris Berendsen

**Affiliations:** 1 Department of Urology, Zuyderland Medical Center, Henri Dunantstraat 5, 6419 Heerlen, The Netherlands; 2 Department of Clinical Chemistry and Haematology, Zuyderland Medical Center, Heerlen, The Netherlands; 3 Department of Microbiology and Infection Control, Zuyderland Medical Center, Heerlen, The Netherlands

**Keywords:** Urine flow cytometry, Urinary tract infections, Urine culture, Urine sediment analysis

## Abstract

**Purpose:**

The aim of our study was to evaluate a procedure in which urine culture was only being performed based on fixed cut-off values of urine sediment analysis with intention to prevent unnecessary negative urine cultures.

**Methods:**

From January 2018 to August 2018, all urine samples from patients visiting the urology outpatient department were analyzed. Urine culture was only performed if urine sediment contained more than 130 bacteria per microliter and/or more than 50 leukocytes per microliter.

**Results:**

In total, 2821 urine cultures with accompanying urine sediments were analyzed. 2098 cultures (74.4%) were defined negative and 723 (25.6%) positive. By adjusting cut-off values of sediment analysis > 20 per microliter or bacteria more than 330 per microliter, 1051 cultures would have been saved with an estimated cost reduction of € 31.470. Eleven clinically relevant urine cultures would have been missed (1%).

**Conclusion:**

Using cut-offs values leads to a notable decrease of the total number of urine cultures. According to our analysis, adjusting cut-off values could result in 37% less urine cultures and almost 50% less negative cultures. Hereby, unnecessary cost can be prevented [in our department estimated €31.470 in eight months (€ 47.205/year)].

## Introduction

Urinary tract infection (UTI) is the second most common infection in first-line health care and the fourth most common infection in second-line health care. A positive urine culture combined with clinical signs of infection is considered as gold standard for urinary tract infections [1]. However, urine cultures are time-consuming, demanding a significant proportion of workload in microbiology laboratories and a significant healthcare expense. More importantly, many of the cultures appear to be negative [2, 3]. Urine sediment analysis is proven effective in reducing number of unnecessary urine cultures [4]. Urine flow cytometry systems are able to rapidly and accurately count the number of white blood cells, bacteria and red blood cells, what can be used to predict bacterial growth in urine cultures. White blood cells and bacteria are both good measures for the screening of UTI. The optimal cut-off values to rule out negative urine cultures are still a matter of debate [2]. The aim of our study was to evaluate a procedure in which urine culture was only processed based on fixed cut-off values of automatized urine sediment analysis with intention to prevent unnecessary negative urine cultures.

## Methods

This is a single-center retrospective observational study. From January 2018 to August 2018, all urine samples from a population of patients visiting the urology outpatient department were analyzed. Midstream urine samples were collected in a sterile vacuum container for urine sediment analyses and urine cultures. Samples from indwelling catheters or clean intermittent catheterization were not included. Urine sediment analysis was performed using the SediMAX FL analyzer (Menarini Benelux, Valkenswaard, The Netherlands), which is an automatic microscopy urine sediment analyzer. Bacterial growth was divided into three groups: less than 130 bacteria/microliter (negative), 130–330 bacteria/microliter (+) or more than 330 bacteria/microliter (+ +). Urine culture was only performed if urine sediment contained more than 130 bacteria/microliter and/or more than 50 leukocytes/microliter. Urine culture was defined negative if there was no bacterial growth at all, growth of ‘mixed contaminants’, < 10e5 colony forming units (cfu)/ml growth of non-classic uropathogen, ≥ 10e5 cfu/ml growth of non-classic uropathogen but with < 20 leukocytes per microliter in sediment analysis and no need for antibiotics, or ≤ 10e4 cfu/ml classic uropathogen with < 20 leukocytes per microliter in sediment analysis and no need for antibiotics. All other urine cultures were considered to be positive. Statistical analysis (namely descriptive statistics) was performed using SPSS version 26.0.

## Results

In total, 2821 urine cultures with accompanying urine sediments were analyzed, from which 2098 (74.4%) were defined negative and 723 (25.6%) positive. 48.4% of urine samples were collected from female patients and 51.6% from male patients. Table 1 shows an overview of the results of urine cultures. Note that only 723 of 865 samples with bacterial growth are finally defined as positive (based on number of microbes and type of pathogen, see also section ‘methods’).

**Table 1:** Overview results urine cultures

Result urine culture	Number (total = 2821)	Percentage (%)
No bacterial growth	1356	48.1
Mixed contaminants	600	21.3
Bacterial growth	865	30.7

From the samples with bacterial growth almost half of the samples showed growth of E. Coli (47.2%). An overview of bacterial growth is shown in Table 2.

**Table 2:** Overview of bacterial and yeast growth

Name of bacterium	Number (total = 865)	Percentage (%)
*Escherichia coli*	408	47.2
*Klebsiella pneumoniae*	85	9.8
*Enterococcus faecalis*	70	8.1
*Proteus mirabilis*	49	5.7
*Beta hemolytic streptococcus B*	44	5.1
*Staphylococcus epidermidis*	28	3.2
*Pseudomonas aeruginosa*	22	2.5
*Aerococcus urinae*	16	1.8
*Staphylococcus aureus*	14	1.6
*Klebsiella oxytoca*	11	1.3
*Enterobacter cloacae*	9	1
*Actinobaculum schaalii*	8	< 1
*Candida* species	4	< 1
Other	90	10.4

We calculated sensitivity and specificity of different cut-off values (Table 3).

**Table 3:** Examples of different urine sediment analysis cut-off values with sensitivity and specificity

	Sensitivity (%)	Specificity (%)
Leukocytes > 75 OR bacteria ≥ ‘ + ’	99	99
Leukocytes > 50 OR bacteria ‘ + + ’	93	56
Leukocytes > 30 OR bacteria ‘ + + ’	94	52
Leukocytes > 25 OR bacteria ‘ + + ’	95	51
Leukocytes > 20 OR bacteria ‘ + + ’	96	47
(Leukocytes > 20 AND bact ≥ ‘ + ’) OR (leukocytes > 100 AND bact neg)	89	64
(Leukocytes > 20 AND bact ≥ ‘ + ’) OR (leukocytes > 50 AND bact neg)	90	62

If cut-off values of sediment analysis were changed to leukocytes > 20 per microliter or bacteria more than 330 per microliter (+ +) and adjusted to the current database, 1019 urine cultures would be correctly negative and, thus, unnecessarily performed (Table 4).

**Table 4:** Adjustment of new cut-off value (leukocytes > 20 OR bacteria ‘ + + ’) to current database

	Urine culture negative	Urine culture positive	Total
Urine sediment negative	1019	32	1051
Urine sediment positive	1079	691	1770
Total	2098	723	2821

On the other hand, 32 positive urine cultures would have been missed. Among these cultures, 19 cultures (59.4%) showed growth of Escherichia coli, 5 (15.6%) Enterococcus faecalis, 3 (9.4%) Proteus mirabalis and 5 (15.6%) various bacteria. However, from those 32 ‘missed’ urine cultures in 21 cases, no treatment with antibiotics or other adjustment was needed. Only 11 cultures appear to be clinically relevant since treatment was given 6 times for symptomatic UTI and 5 times as prophylaxis before an urological procedure.

In Table 5, an estimation is shown of the potential cost reduction. If in retrospect, a cut-off of leukocytes > 20 per microliter or bacteria > 330 per microliter were used in our dataset, 1051 cultures (37% of all cultures) would have been saved in 8 months with an estimated cost reduction of € 31.470. When we keep in mind that only 11 urine cultures were clinically relevant, 93 urine cultures (with a total estimated value of €2785) must be performed in order not to miss one clinically relevant positive urine culture. [5]

**Table 5:** Potential cost reduction with new cut-off values (leukocytes > 20 and bacteria + +)

	Price	Number urine cultures	Cost
Urine cultures negative	€ 24,66	957	€ 23.600
Urine cultures positive			
Gram-positive	€ 68,87	38	€ 2.617
Gram-negative	€ 90,11	53	€ 4.776
Both	€ 158,98	3	€ 477
Total		1051	€ 31.470

## Discussion

Reducing unnecessary health care costs is important to improve quality of health care. Moreover preventing unnecessary antibiotic treatment is key in reducing antibiotic resistance. Sediment analysis and urine cultures are frequently performed in the urology outpatient department, as well as in other hospital departments and general practices. Cut-off values of sediment analysis are still a matter of debate. The large heterogeneity in study designs makes it challenging to draw conclusions [2]. De Rosa et al. states that each laboratory should establish its own optimal cut-off values based on the population and different criteria for positive urine cultures, although these values should be periodically reviewed [3]. In this study, we presented a practical insight of the benefits when adjusting cut-off values in our laboratory.

The aim of the study was to prevent unnecessary urine cultures. How many positive urine cultures are acceptable to miss to prevent negative urine cultures is of course a subject of debate, and different clinicians might come to varying conclusions. Our cut-off values, mostly on bacteriuria, do not correspond with other recommended cut-off values from studies analyzing SediMAX FL. Sterry-Blunt et al. recommend a combined cut-off values of 11 leukocytes/microliter and 167 bacteria/microliter [6] and Inigo et al. proposed cut-off values of 18 leukocytes/microliter and 97 bacteria/ microliter [7]. Both studies conclude a stronger correlation for leukocytes than for bacteriuria. The latter is in accordance with our results.

The conflicting data on cut-off values might be explained by first of all the fact that our dataset was already a result of quite high fixed cut-off values (> 130 bacteria/microliter and/or > 50 leukocytes/microliter) and secondly by different definitions of positive urine cultures. As summarized by Chang et al., there is a large variation in reference standards in studies about urine flow cytometry. Colony forming units’ cut-offs to determine a positive urine culture range from 10^2^ to 10^5^ cfu/ml [2]. We carefully discussed the determination of urine culture as either positive or negative with a microbiologist. Because we were specifically interested in the clinical approach and consequences, we chose a more practical distinction rather than solely laboratory cut-off values.

Since our study population were merely patients from the urology department, it must be noted that our results might not be applicable for patients from other departments or general practice. Our hospital laboratory roughly performs 38,000 urine cultures every year, collected from all departments and clinical practice. If a similar reduction in number of urine cultures could be achieved then even a greater amount of unnecessary costs could be prevented.

A limitation of the study is the process of urine sample collection. As stated in the methods section we used midstream urine samples. Despite patient instructions, we cannot be certain that all patients performed proper urine collection. Second, catheterized samples or samples from indwelling catheters were not included in the database, but we cannot rule out with certainty incorrect orders.

Adjusting cut-off values remains an ongoing process. We chose quite high cut-off values (especially for bacteriuria) comparing to the literature, knowing that we will miss a very small amount of clinically relevant positive urine cultures (1%). In the future, we need to evaluate the impact of that on our clinical care.

## Conclusion

Using cut-offs values leads to a notable decrease of the total number of urine cultures compared to performing urine cultures without sediment analysis. According to our analysis, adjusting cut-off values (leukocytes > 20/µl or bacteria > 330/µl) could result in 37% less urine cultures and almost 50% less negative cultures. Hereby, unnecessary cost can be prevented (in our department roughly estimated €31.470 in 8 months).
